# Exploring pig trade patterns to inform the design of risk-based disease surveillance and control strategies

**DOI:** 10.1038/srep28429

**Published:** 2016-06-30

**Authors:** C. Guinat, A. Relun, B. Wall, A. Morris, L. Dixon, D. U. Pfeiffer

**Affiliations:** 1 Department of Production and Population Health, Royal Veterinary College, Hawkshead Lane, Hatfield, Hertfordshire, AL9 7TA, United Kingdom; 2 The Pirbright Institute, Ash Road, Pirbright, Surrey, GU24 0NF, United Kingdom; 3 Centre de coopération international en recherche agronomique pour le développement (CIRAD), UPR AGIRs, Campus international de Baillarguet, F-34398 Montpellier, France; 4 Department of Epidemiological Sciences, Animal and Plant Health Agency (APHA) Weybridge, Woodham Lane, Addlestone, Surrey, KT15 3NB, United Kingdom

## Abstract

An understanding of the patterns of animal contact networks provides essential information for the design of risk-based animal disease surveillance and control strategies. This study characterises pig movements throughout England and Wales between 2009 and 2013 with a view to characterising spatial and temporal patterns, network topology and trade communities. Data were extracted from the Animal and Plant Health Agency (APHA)’s RADAR (Rapid Analysis and Detection of Animal-related Risks) database, and analysed using descriptive and network approaches. A total of 61,937,855 pigs were moved through 872,493 movements of batches in England and Wales during the 5-year study period. Results show that the network exhibited scale-free and small-world topologies, indicating the potential for diseases to quickly spread within the pig industry. The findings also provide suggestions for how risk-based surveillance strategies could be optimised in the country by taking account of highly connected holdings, geographical regions and time periods with the greatest number of movements and pigs moved, as these are likely to be at higher risk for disease introduction. This study is also the first attempt to identify trade communities in the country, information which could be used to facilitate the pig trade and maintain disease-free status across the country in the event of an outbreak.

Trade-related movements of animals represent an important means for disease spread between farm premises^1^. Recent advances in computational power and availability of higher quality data have made it possible to apply network analysis to national level animal production and health data. This has led to improved quantification and description of movements between premises for the study of disease spread^2^,^3^,^4^,^5^,^6^,^7^,^8^,^9^. The resulting information demonstrates the heterogeneity of contacts in animal populations, in contrast to the homogeneity which has previously been assumed in the design of surveillance and control strategies^10^. Network analysis characterises the relationships between premises (defined as ‘nodes’) on the basis of any links resulting from animal movements (defined as ‘edges’ which can be weighted based on the number of movements or number of pigs moved per movement or in total). It is relevant for characterising the temporal and spatial variability of animal contact patterns^11^. The approach has recently been used in disease control and surveillance, for understanding the dynamics of past epidemics^3^,^8^, assessing the impact of movement bans^12^,^13^ and predicting the size of epidemics^14^,^15^.

Great Britain (GB) has one of the largest pig industries in the European Union (EU), producing over 8 million pigs for human consumption per year^16^. In recent years, total pig meat exports from the United Kingdom (UK) to EU and non-EU countries were estimated at over 200,000 tonnes per year, valued at over £300 million GBP^17^. Future pig disease outbreaks could have a significant socio-economic impact on British pig producers, especially through temporally restricting or preventing access to international trade in pigs and pig products. Stricter regulations on animal movement reporting were implemented in response to the 2001 foot-and-mouth disease (FMD) epidemic in the UK, and retrospective analyses indicated that disease spread was largely influenced by animal movements and mixing at market premises^18^,^19^. The importance of contact networks in the ongoing outbreaks of bovine tuberculosis (bTB) in the UK has also been highlighted^20^. The severe impact of recently reported pig disease outbreaks in Europe and North America, including African swine fever^21^ and porcine epidemic diarrhoea (PED)^22^, have emphasised the need to better understand the British pig contact network, as this will allow more effective risk management of movements that may facilitate disease transmission in the event of a disease outbreak.

A number of descriptive network measurements are used in the analysis of animal movements^23^,^24^. Premises with a high out-degree (number of outgoing movements) can act as important sources for disease spread; whereas premises with a high in-degree (number of incoming movements) are likely to be at risk of disease introduction. A scale-free network, in which in- and out-degree distributions fit a power law distribution, may allow diseases to spread more quickly than a network with less highly connected premises. A small-world network, characterised by the presence of distinct clusters, connected to topologically distant clusters through a small number of connections, may also allow for the rapid spread of diseases and facilitate persistence compared with a random network. In a transmission network, the size of the giant strong component (a group of premises which are all directly or indirectly connected with each other) may provide an indication of the likely size of a potential epidemic in the absence of intervention. Understanding of animal movement networks in terms of these network features can support compartmentalisation approaches for disease control, which are encouraged by the World Organisation for Animal Health (OIE) and the World Trade Organisation (WTO)^25^,^26^ and may support the possibility of continued international trade even in the event of a disease outbreak in a particular country or region.

A previous study investigated pig movements in the UK over a 12-month period, using pig surveillance data from three Quality Assurance (QA) schemes^27^. However, compared with the RADAR database, QA records lack information on pig movements involving small and semi-professional holdings, gathering areas and markets; and these types of premises may indeed have a particularly strong influence on disease transmission^13^,^18^. Other authors have also highlighted that farm connectivity may have been underestimated as a result of non-reported movements (due to farmer self-reporting and recall bias, non-inclusion of non-QA registered holdings)^27^. Consequently, there are significant knowledge gaps in relation to the overall structure of pig movement networks in Great Britain, which compromises the ability to design effective risk-based disease surveillance and control strategies.

The aim of this study was to explore the pig movement networks throughout England and Wales with a view to characterising spatial and temporal patterns, monthly network topology and trade communities. This analysis was based on data covering 5 years (2009–2013) which had been extracted from the RADAR database. The results from this study will make an important contribution to the knowledge base informing the design of risk-based disease surveillance and control programmes for the British pig industry.

## Results

### Data description

The British pig industry is organised in a pyramidal structure^28^. At the top, nucleus farms provide purebred sows and boars to multiplier farms, which produce crossbred pigs and gilts to supply breeding farms. At the bottom, commercial production farms produce pigs for slaughter. Overall, 61,937,855 pigs were involved in 872,493 movements that occurred during the 5-year study period. Of these, 3.4% of movements, representing 4.4% of pigs, were excluded from the analysis due to missing data regarding the type of premises (97.6% of these) or the geographical coordinates (2.4% of these).

#### Number of premises

Pig holdings accounted for most of the premises (up to 98.6%) in the movement database (See Supplementary Table S1). In 2009, there were 22,310 pig holdings, 217 gathering areas, 174 slaughterhouses and 16 markets that reported moving pigs. From 2009 to 2011, pig holding numbers increased by 13.8% while from 2011 to 2013, they decreased by 26.4%. The number of gathering areas (i.e. area of common land used for grouping pigs for temporary time periods before they are moved to other premises), slaughterhouses and markets also first increased (from 2009 to 2011) and then decreased (from 2011 to 2013) over the study period. The distribution of herd sizes was right-skewed, with 2,115 (5%) holdings identified as “large” (more than 5,000 pigs), 1,514 (4%) as “medium” (between 500–5,000 pigs) and 37,645 (91%) as “small” (less than 500 pigs).

#### Characterisation of movements between premises

Overall, most of the movements originated from pig holdings (96.1%) and were directed to slaughterhouses (68.3%) and to other pig holdings (23.7%). Similarly, most pigs were moved from pig holdings (98.8%) to slaughterhouses (56.2%) and to other pig holdings (43.5%).

Table 1 describes the movements between premises over the 5-year study period. The highest number of holding-to-holding movements was observed between large holdings (49.7%), although movements also frequently occurred from small to large pig holdings (34.9%). However, large-to-large holding movements involved the majority of pigs transported (96.2% vs. 1.1%). Large holdings were responsible for the majority of movements and pigs moved to markets (58.4% and 51.3%, respectively) and to slaughterhouses (50.2% and 91.5%, respectively). They also moved the majority of pigs to gathering areas (40.6%). Small and medium holdings were involved in a lot of movements to slaughterhouses (32.1% and 15.8%) and gathering areas (36.7% and 17.8%, respectively), but pigs were in smaller batches. Large holdings received the majority of movements and pigs from gathering areas (24.0% and 18.2%, respectively) and markets (32.9% and 47.5%, respectively).

The distribution of geographical distances covered per movement during the 5-year study period was mainly right-skewed (Table 1), with a large number of movements (75%) covering short distances (less than 65 km) whereas a small number of movements (5%) were over long distances (more than 175 km, with a maximum of 767 km).

#### Spatial and temporal movement patterns

**Figure 1:**
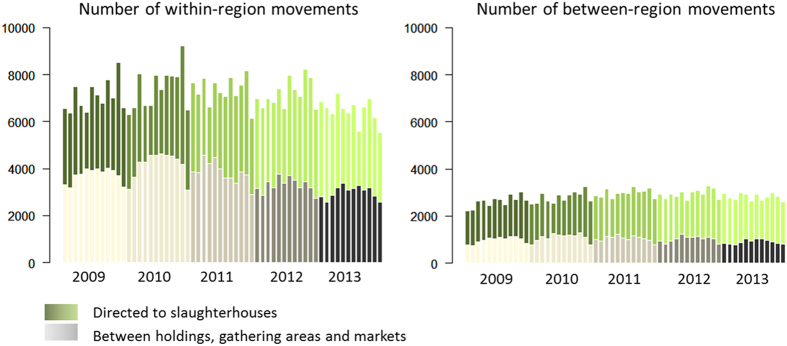
Temporal distribution of the number of within- and between-region movements occurring between pig holdings, gathering areas and markets, and directed to slaughterhouses, over the 5-year study period (2009–2013) in England and Wales.

Figure 1 shows the distribution of the within- and between-region movements of pig batches per month directed to slaughterhouses and those among pig holdings, gathering areas and markets over the 5-year study period. The majority of movements (73.6%) were observed within NUTS-defined regions, using NUTS level 1 for England (e.g. East Anglia, East Midlands, North East England, North West England, South East England, South West England, West Midlands, Yorkshire and the Humberside) and NUTS 2 level for Wales (e.g. Central Wales, North Wales and South Wales), accounting for 61.8% of the pigs moved over the 5-year study period. South West England, Yorkshire and Humberside and East Anglia were the regions with the highest number of movements taking place within their boundaries (23.0%, 21.3% and 17.5%, respectively). From 2009 to 2010, the overall number of movements increased by 6.9% associated with an 7.5% increase in number of pigs moved. From 2010 to 2013, the overall number of movements was down by 14.7%, with a 12.2% increase in the number of pigs moved. The same seasonal pattern of movements was repeated every year, with most movements among pig holdings, gathering areas and markets occurring from late spring to autumn (i.e. from May to September). Most movements directed to slaughterhouses took place in autumn (i.e. from September to November).

**Figure 2:**
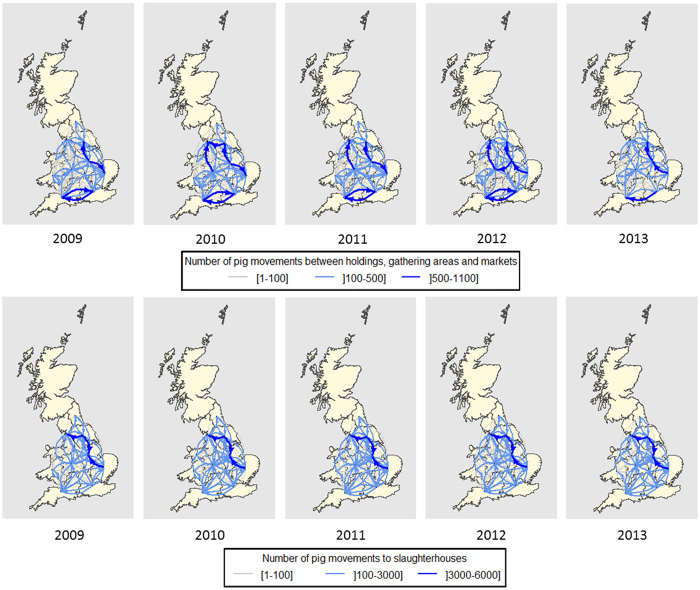
Spatial representation of the number of between-region movements in each year, occurring between pig holdings, gathering areas and markets, and directed to slaughterhouses, over the 5-year study period (2009–2013) in England and Wales. This map was created using R statistical software^53^ (version 3.0.2).

Figure 2 shows the distribution of the between-region movements of pig batches among pig holdings, gathering areas and markets and those directed to slaughterhouses over the 5-year study period. Every year, East Midlands and South East England were the major senders of pigs among pig holdings, gathering areas and markets (20.2% and 11.7%, respectively) and Yorkshire and Humberside and South West England were the major receivers (17.4% and 11.3%, respectively). Every year, Yorkshire and Humberside and East Anglia were the major senders of pig movements to slaughterhouses (32.0% and 14.9%, respectively) and East Midlands and North West England were the major receivers (27.5% and 21.9%, respectively).

### Network analysis

**Figure 3:**
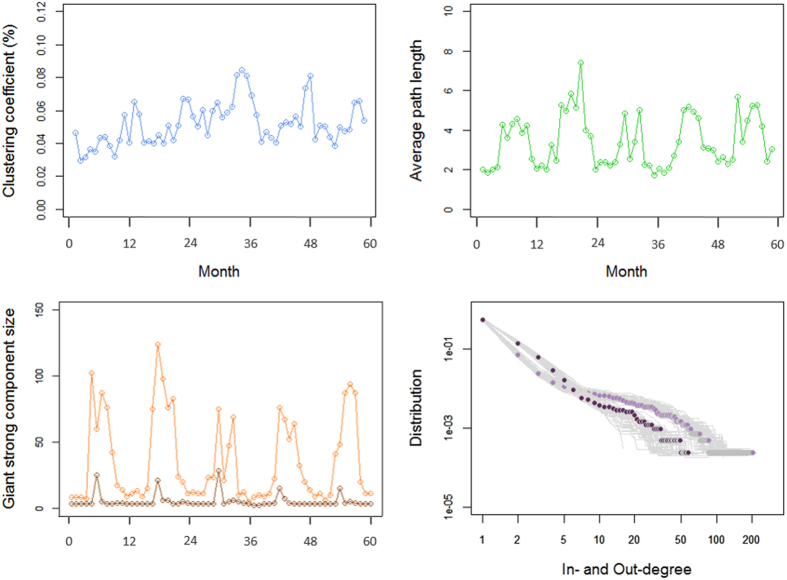
Temporal distribution of the descriptive network parameters: clustering coefficient, average path length, giant strong component size (orange: pig holdings, gathering areas, markets; brown: pig holdings and markets), in-degree (light purple) and out-degree (dark purple), characterising pig movements over the 5-year study period (2009–2013) in England and Wales.

Figure 3 shows the distribution of the network parameters over the 5-year study period, obtained from networks constructed on a monthly basis. The monthly median APL (Table 2) obtained from the static and non-directed networks, ranged from 2.39 to 3.85, meaning that any two premises were separated by approximately 2 to 4 movements every month. The monthly median GSC size (Table 2) varied from 15.5 to 22 nodes, meaning that between 15 and 22 premises were involved in the GSC every month. In most months, randomly generated networks showed a larger APL and a lower CC (Table 2) compared to the observed networks. The APL and the GSC size distributions varied within years, marked by an increase from late spring to autumn. The exclusion of gathering areas resulted in minimising the GSC size, with an 85% decrease in the median size. The in-degree and out-degree distributions were heavy-tailed and approximated a power law distribution with a median exponent of 4.6 and 3.7, respectively.

**Figure 4:**
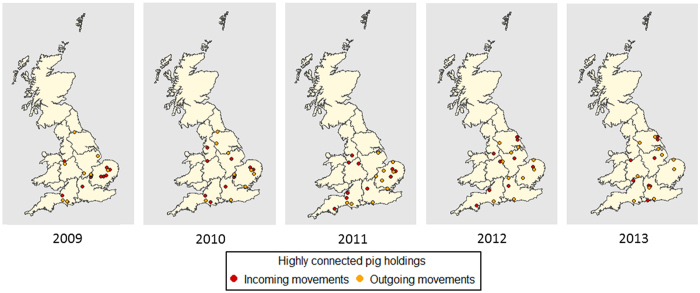
Spatial representation of the ten most highly connected pig holdings in each year, over the 5-year study period (2009–2013) in England and Wales. This map was created using R statistical software^53^ (version 3.0.2).

The ten most highly connected pig holdings, in terms of numbers of incoming and outgoing movements, were identified over the 5-year study period (Fig. 4). They accounted for 0.2% of all pig holdings, for 2.6% and 12.2% of all outgoing and incoming movements of pig holdings, respectively. In 2013, 50.0% of the highly connected holdings were identical to those identified in 2012. 25.0%, 40.0% and 25.0% were identical to those in 2011, 2010 and 2009, respectively. They were quite homogeneously distributed over time, but in 2013 spatially more concentrated in Yorkshire and Humberside and South East England in contrast to the past four years in East Anglia and South West England.

**Figure 5:**
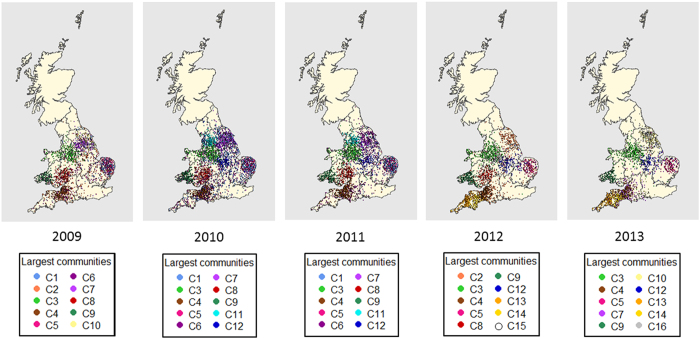
Spatial representation of the ten largest trade communities in each year (coloured according to community) including pig holdings, gathering areas and markets, over the 5-year study period (2009–2013) in England and Wales. This map was created using R statistical software^53^ (version 3.0.2).

The communities, in terms of numbers of holdings, gathering areas and markets were identified based on yearly networks over the 5-year study period (See Supplementary Table S2). In total, there were from 3,220 to 3,911 communities amongst the 14,726 to 21,316 premises, depending on the year. The modularity measure increased from 0.739 to 0.786 from 2009 to 2013. The ten largest communities represented 0.3–0.4% of these communities and 16.2–24.5% of these premises depending on the year (See Supplementary Table S2). In 2013, 44.3% of the premises belonging to the ten largest communities were identical to those identified in 2012. There were 32.4%, 34.2% and 23.7% identical to those in 2011, 2010 and 2009, respectively. Most of the ten largest communities were closely related to specific NUTS regions (NUTS level 1 and level 2 for England and Wales, respectively) (Fig. 5). South West England comprised three of the ten largest communities during the study period: community C4 from 2009 to 2013 and communities C13 and C14 from 2012 to 2013. East Anglia comprised two of the ten largest communities: C1 from 2009 to 2012 and community C5 from 2009 to 2013. Yorkshire and Humberside comprises several communities that also extended into other regions, including C2, C7, C10, C11 and C16. Other communities spanned several regions.

## Discussion

This study presents an analysis of the space-time patterns of movements of pig batches and total pigs moved per year between pig holdings, gathering areas, markets and slaughterhouses in England and Wales, based on data covering a 5-year period (2009–2013) extracted from the RADAR database. Results indicate an overall decline in the number of movements of pig batches from 2010 onwards, and in the number of pig holdings from 2011, while the total number of pigs moved during the study period increased. This can be explained by changes in pig farm size distribution, in particular an increase in the number of large pig farms and the disappearance of small farms, as previously reported by the Agriculture and Horticulture Development Board (AHDB) Pork, and is in agreement with the general trend observed in other pig-producing EU countries^28^,^29^. This probably reflects the need for pig production to become more cost-effective through intensification, which is also consistent with the observation of fewer movements comprising larger batches of pigs. Changes in the number of pig holdings are also likely to be related to pig meat and feed market price trends. For example, there was a significant global rise in the cost of pig feed in 2012, which had a considerable effect on the structure of Great Britain’s pig industry and impacted the livelihoods of many farmers^30^. However, it is worth noting that there may also be an effect of lack of compliance with the recording of movements rather than an actual change in the number of premises. For example, a recent survey conducted amongst small pig holding and pet pig owners in England showed that around 17% of the respondents were not aware of the movement reporting requirements^31^. However, this is not likely to impact greatly on the results of this study, as most of them reported having a small number of pigs (median 4) that were mostly bred for own consumption (44.6%) or kept as pets (37.0%).

Results show that most movements of pig batches occurred within regions, particularly within the boundaries of East Anglia, Yorkshire and Humberside and South West England. Accordingly, movements mainly occurred over short geographical distances, with 50% and 95% of them covering less than 31 km and 65 km, respectively, with a maximum of 767 km. These findings suggest that disease outbreaks are more likely to remain restricted to a local geographical scale, such as within regional boundaries. Every year, the majority of between-region movements from premises (holdings, gathering areas and markets) to slaughterhouses occurred among the largest commercial pig-producing areas, i.e. from Yorkshire, Humberside and East Anglia to East Midlands and North West England. Every year, the majority of between-region movements among holdings, gathering areas and markets were directed from East Midlands and South East England towards Yorkshire and Humberside and South West England. These results can be also explained by the higher density of large commercial holdings located in the regions of East Anglia, Yorkshire and Humberside, as well as the higher density of small size units (such as backyard, pedigree and show pigs) in South West England. This suggests that there are geographical pig movement channels which it may be useful to target as part of disease surveillance and control efforts.

There were a higher number of small sized pig holdings than medium and large. However, movements of pig batches between holdings were mainly reported among those identified as large, and these accounted for the majority of pigs. Large pig holdings were closely connected to markets and to a lesser extent to gathering areas. Small and medium sized pig holdings also accounted for a high number of batch movements to large sized pig holdings, to gathering areas and slaughterhouses but with a smaller number of pigs. This highlights additional risks of disease spread within the pig industry, since the holdings with smallest pig numbers are more likely to have poor biosecurity than large ones. For example, around 23% of small pig holding or pet pig owners interviewed in England have reported using swill to feed their pigs^31^. Such results have also been noted for the Scottish pig network^32^. These findings emphasise the importance of appropriate biosecurity and disease awareness at both large and small premises. Efforts should be focused towards addressing the significant role that all industry stakeholders (such as pedigree pig breeders, commercial farmers, processors, etc.) play in swine disease surveillance.

Results of the data analysis revealed a higher number of movements of pig batches from late-spring to autumn, linked with the breeding and production cycle as pigs moved from the growing to finishing phase, and with periods of increased slaughtering, particularly before Christmas and Easter. This seasonal pattern has also been observed in Spanish, Swedish and Scottish pig movement networks^7^,^32^,^33^, and suggests that outbreaks which occur during these periods (particularly when directed to pig holdings, gathering areas and markets), would have the potential to spread more widely. Therefore, intensifying surveillance during this period may be particularly effective for disease prevention and control.

The network exhibits small world properties, as also has been described for the Danish, Italian, Spanish and French pig movement network and in previous analyses of UK pig movement data^2^,^27^,^34^. This indicates that most nodes are not directly connected to each other but can be reached through a small number of connections. It allows for disease to spread to more distant clusters and facilitates persistent infection in the pig population compared with a random network^35^. The network exhibits scale-free properties, consistent with previously studied networks for the Danish, Belgian, Swedish and French pig industries^2^,^7^,^34^,^36^,^37^. This topology indicates the presence of highly connected nodes (‘hubs’) potentially, acting as super-spreaders, through which disease could spread more rapidly than in random networks^38^. Up to 50.0% of the ten most influential hubs in this network in each year were the same between 2012 and 2013. As a result, surveillance and biosecurity campaigns targeted at the nodes within these highly connected hubs may result in more timely detection of outbreaks and reduction in the size of outbreaks. Moreover, these hubs could be targeted for dissemination of up-to-date information about disease surveillance and control or any relevant knowledge that enhances the effectiveness of disease prevention and control amongst pig industry stakeholders.

When considering the GSC size, gathering areas also appeared to have an important potential role in disease transmission, as has similarly been observed previously for cattle movement networks in GB, France and Denmark^6^,^13^,^39^. Although the GSC size may be an overestimate of a likely epidemic size^40^, it is useful information for determining the number of potentially exposed farms^41^. Again, enhancing surveillance and biosecurity at these premises is likely to have significant impact in terms of minimising the extent of a potential epidemic. The ten largest communities accounted for up to 24.5% of all premises. They were broadly associated with particular geographical regions. Up to 44.3% of the community premises remained unchanged between 2012 and 2013. These community borders could eventually be used for defining trade compartments in England and Wales, although they should first be further investigated - particularly regarding other potential pathways of disease introduction into such communities - in order to increase confidence in the effectiveness of compartmentalisation as a strategy for controlling disease epidemics while minimising disruption to trade^42^.

The RADAR database allowed access to detailed information on pig movements in England and Wales, permitting exploratory analyses of the contact network. Amongst the movement records in the database, less than 4% had missing data on geographical coordinates or premises type and these were excluded from the computations. This will have resulted in some degree of underestimation of the number of contacts between premises. Some premises were also incorrectly georeferenced. Twenty-one movements originating from slaughterhouses were recorded; these might be due to misclassification of the type of premises or recording of illegal movements. Underreporting of pig movements by pig owners still remains possible, however they are difficult to quantify due to lack of data. Around 17% of small pig holding and pet pig owners interviewed in England reported not being registered with the Department for Environment, Food and Rural Affairs (Defra) despite it being compulsory^31^. These findings indicate that further efforts should be made to optimise the accuracy of the movement records, so that the influence of bias can be further reduced.

A compulsory 20-day standstill period is implemented in the UK, restricting movements of pigs from premises for 20 days following the introduction of any new pigs onto a premises^43^. This should increase the likelihood of disease detection before moving animals, reducing the risk of disease spread between premises. However, disease spread remains possible through other means including movements of potentially contaminated vehicles, equipment and animal workers. For example, several studies have demonstrated that the movement of transport lorries may play a major role in the transmission of diseases such as PED, classical swine fever (CSF) and *Salmonella* infection when adequate cleaning and disinfection measures are not implemented between premises, particularly when groups of pigs originating from different premises are moved by a single vehicle^44^,^45^,^46^. In particular, those coming back from the slaughterhouses have shown a great potential for transmission of diseases^41^,^47^. However, information regarding lorry movements was not available, reducing the potential for more in-depth analyses. Moreover, no further differentiation was possible among pig holding premises, preventing the differentiation of hobby pig keepers from professional producers in this dataset, other than by using the size of premises as a proxy. Distances were measured as Euclidean distance between premises as an approximation of actual road travel distance. A more accurate alternative would be to introduce GPS (Global Positioning System) recording devices onto pig lorries. These are increasingly being used in private and public transportation vehicles including courier vehicles, ambulances and buses to geographically locate vehicles and precisely track their movements^48^,^49^. While there is significant scope for further improving the movement reporting system in the UK, the analyses presented here have demonstrated how it is already possible to produce useful outputs that can inform the design of risk-based disease surveillance and control programmes.

## Methods

### Data collection

Pig farmers are required by the UK government to pre-notify movements from their farms, and to report movements onto their farm within three days of the movement having taken place^43^. All pig movement data are then collected by the Rapid Analysis and Detection of Animal-Related Risks (RADAR) team at the Animal and Plant Health Agency (APHA) and analysed through the RADAR analytical system^50^. Data on pig movements in England and Wales were extracted from the RADAR database, covering the time period from January 1^st^, 2009 to December 31^st^, 2013. Each movement record - defined as a movement of a batch of pigs between two different premises on the same day - consisted of the date of movement, the departure premises identifier, the locations of the departure and arrival premises, the type of departure and arrival premises and the number of pigs moved. The premises’ geographical locations were expressed using easting and northing coordinates (i.e. x and y values expressed in metres) and reflected the location of either animal or owner housing. The different types of premises were: pig holding, slaughterhouse, gathering area and market. Premises entitled ‘pig holding’ included all types of premises where pigs are kept, including households with pigs kept as pets, pig shows and pig farms. Premises entitled ‘gathering’ were defined as an area of common land used for grouping pigs for temporary time periods before being moved to other premises. Premises entitled ‘market’ also included showgrounds. No information was available on transport lorry movements or farms’ specific characteristics (e.g. herd size and type of production). Movements with missing data for any of the above variables were excluded from the analysis. This work contains public sector information licensed under the Open Government Licence v3.0. To view a copy of this licence, visit http://www.nationalarchives.gov.uk/doc/open-government-licence/version/3/.

### Descriptive analysis

Pig holdings were classified into three categories: “large”, “small” and “medium” if they had moved more than 5,000 pigs, less than 500 pigs, and between 500–5,000 pigs, respectively during the 5-year study period. This was based on the typical size distribution of commercial pig holdings^51^. Premises were geographically grouped according to their NUTS (Nomenclature of Units for Territorial Statistics) region^52^. Regions were defined using NUTS level 1 and level 2 for England (e.g. East Anglia, East Midlands, North East England, North West England, South East England, South West England, West Midlands, Yorkshire and the Humberside), and Wales (e.g. Central Wales, North Wales and South Wales), respectively. Distances covered by movements were calculated using Euclidean distance (e.g. straight-line distances). Descriptive analyses (median, mean, interquartile, maximum and graphical) and maps were computed using the R statistical software^53^ (version 3.0.2).

### Network analysis

Pig movements were represented as a directed network, considering each premises as a node and a movement of batches of pigs between two premises as an edge. Pig holdings, gathering areas and markets were included in the network analyses, as mixing of pigs at markets and gathering places has been suggested to be an important factor for disease spread^13^,^18^. Slaughterhouses were excluded as they were assumed to be dead-ends in terms of disease transmission. Data were aggregated temporally by month to estimate the general network properties, since this represents a likely epidemic duration prior to detection of important swine diseases (such as CSF and FMD) and implementation of movement bans^18^,^44^. This time period has been frequently used in previous pig network analyses^7^,^36^,^37^, and thereby will facilitate between-country comparisons. Data were aggregated by year to detect communities. Weights were assigned to each edge according to the number of pig batches moved between two nodes (premises) within each period (each month for the general network properties and each year for the communities).

Descriptions of the network terminology are outlined in Table 2. To characterise the network topology, the clustering coefficient (CC), the average path length (APL) and the degree measures were computed for each month of the 5-year study period, and their distributions were plotted over time. The network was examined for small-world and scale-free properties. A small-world network is characterised by nodes with a higher CC and a smaller APL compared to the maximum CC and minimum APL derived from a set of randomly generated networks (i.e. 100 networks randomly simulated, using the same number of nodes and edges)^54^,^55^. A scale-free network is characterised by a right skewed, long-tailed, power law distribution of the number of edges (out-degree and in-degree) to nodes^56^. Parameters of the fitted power law distribution were calculated using statistical approaches described in Clauset *et al.*^57^. The size of the giant strong component (GSC) was computed for each month of the 5-year study period and its distribution was plotted over time. The premises type (pig holding, gathering area or market, removed one at a time) whose removal minimises the size of the GSC and therefore the size of an epidemic^58^ was considered as playing an important role in transmitting diseases. Trade communities were identified in the network for each year using a random walk algorithm^59^ and were mapped individually. Network analyses were computed and maps were created in R statistical software^53^ (version 3.0.2) using the ‘igraph’ and ‘rgdal’ packages^60^,^61^.

## Additional Information

**How to cite this article**: Guinat, C. *et al.* Exploring pig trade patterns to inform the design of risk-based disease surveillance and control strategies. *Sci. Rep.* **6**, 28429; doi: 10.1038/srep28429 (2016).

## Supplementary Material

Supplementary Information

## Figures and Tables

**Table 1:** Number of movements, pigs moved and kilometres covered between pig holdings, gathering areas, markets and slaughterhouses over the 5-year study period (2009–2013) in England and Wales.

Types of movements	Number of movements		Number of pigs moved		Distance (km)	
	Total	Proportion (%)	Total	Proportion (%)	Median (Q1–Q3)	Max
Pig holding						
Small - Small	6,935	3.7	24,869	0.0	19 (8–45)	725
Small – Medium	1,617	0.8	5,517	0.0	16 (9–23)	165
Small – Large	64,887	34.9	295,231	1.1	16 (7–36)	720
Medium - Small	699	0.4	4,109	0.0	20 (9–42)	433
Medium - Medium	490	0.2	7,527	0.0	20 (11–26)	118
Medium - Large	17,908	9.6	666,003	2.4	25 (11–51)	727
Large – Small	18	0.0	2,030	0.0	176 (162–233)	357
Large - Medium	1,082	0.6	38,034	0.2	47 (25–85)	517
Large – Large	92,435	49.7	26,234,194	96.2	41 (16–88)	646
Gathering area						
Small - Gathering	34,043	36.7	256,426	23.1	31 (18–50)	518
Medium - Gathering	16,555	17.8	194,208	17.5	30 (19–52)	497
Large - Gathering	18,953	20.4	449,840	40.6	27 (15–43)	452
Gathering - Small	915	1.0	5,734	0.5	32 (20–50)	627
Gathering - Medium	30	0.0	449	0.0	11 (11–68)	114
Gathering - Large	22,222	24.0	202,346	18.2	35 (20–57)	499
Market						
Small - Market	44	5.6	311	0.5	19 (9–34)	450
Medium - Market	19	2.4	403	0.6	11 (11–11)	27
Large - Market	463	58.4	32,392	51.3	11 (11–11)	85
Market - Small	5	0.6	55	0.0	72 (37–90)	187
Market - Medium	1	0.1	2	0.0	41 (41–41)	41
Market - Large	261	32.9	29,946	47.5	1 (1–12)	192
Slaughterhouse						
Small -aughterhouse	195,131	32.1	699,109	2.0	19 (11–31)	767
Medium -Slaughterhouse	96,089	15.8	1,772,878	5.0	24 (13–43)	723
Large -Slaughterhouse	304,795	50.2	32,318,300	91.5	57 (27–75)	721
Gathering area -Slaughterhouse	10,935	1.8	520,664	1.4	58 (23–253)	715
Market -Slaughterhouse	123	0.0	14,499	0.0	10 (10–10)	110

Pig holdings were classified into three size categories as “small” (less than 500 pigs), “medium” (between 500–5,000 pigs) and “large” (more than 5,000 pigs).

**Table 2:** Network terminology.

Parameter	Definition
Node	Premises (i.e. pig holdings, gathering areas, markets and slaughterhouses which have moved at least one pig during a particular period of time).
Edge	Directed movement of pigs between two premises^11^.
Clustering coefficient (CC)	Probability that two premises that are both in direct contact with the same premises are also directly connected to each other^55^.
Average path length (APL)	Shortest path among two premises averaged over all pairs of premises in the network^55^. A path consists of the number of edges between two nodes.
Degree	Number of incoming (in-degree) and outgoing (out-degree) movements from a premise to other premises^11^.
Giant strong component (GSC) size	Number of premises which are all directly or indirectly connected with each other^4^.
Small-world	Network characterised by high clustering and short path length^55^.
Scale-free	Network where the out-degree and in-degree distributions fit a power law distribution^56^.
Trade community	Subsets of nodes which have significantly more edges (trade connections) than expected by chance^62^. Modularity measure quantifies the quality of the community structure^59^.
