# Supplementary material for: Exploring pig trade patterns to inform the design of risk-based disease surveillance and control strategies
Source: Sci Rep. 2016 Jun 30;6:28429. doi: 10.1038/srep28429 (PMC4928095; doi:10.1038/srep28429)
Supplement: Supplementary Information [file srep28429-s1.pdf]

Title: Exploring pig trade patterns to inform the design of risk-based disease surveillance and control strategies

Author names and affiliations: C. Guinat[1,2], A. Relun[3], B. Wall[1], A. Morris[1,4], L. Dixon[2], D.U. Pfeiffer[1]

[1] Veterinary Epidemiology, Economics and Public Health Group, Royal Veterinary College, Hawkshead Lane, Hatfield, Hertfordshire, AL9 7TA, United Kingdom

[2] The Pirbright Institute, Ash Road, Pirbright, Surrey, GU24 0NF, United Kingdom

[3] Centre de coopération international en recherche agronomique pour le développement (CIRAD), Département ES, UR22, TA C22/E, Campus international de Baillarguet, 34398 Montpellier Cedex 5, France

[4] Department of Epidemiological Sciences, Animal and Plant Health Agency (APHA) Weybridge, Woodham Lane, Addlestone, Surrey, KT15 3NB, United Kingdom

Supplementary Tables

Table S1. Number and type of premises over the 5-year study period (2009-2013) in Great Britain

Year	Pig holding		Gathering area		Slaughterhouse		Market	
	Total	Proportion (%)	Total	Proportion (%)	Total	Proportion (%)	Total	Proportion (%)
2009	22,310	98.2	217	1.0	174	0.8	16	0.0
2010	25,303	98.5	202	0.8	166	0.6	21	0.1
2011	25,879	98.4	218	0.8	178	0.7	22	0.1
2012	22,145	98.6	147	0.7	158	0.7	6	0.0
2013	19,046	98.5	144	0.7	150	0.8	6	0.0

Table S2. Estimates of descriptive network parameters characterising the trade communities over the 5-year study period (2009-2013) in Great Britain

	Year				
	2009	2010	2011	2012	2013
All communities					
Number	3,437	3,224	3,911	3,220	2,756
Nodes	18,611	21,316	21,095	17,301	14,726
Edges	28,260	32,268	30,645	25,310	21,716
Modularity	0.739	0.743	0.753	0.766	0.786
Holding	18,379	21,105	20,867	17,158	14,583
Gathering	215	192	206	139	140
Market	17	19	22	4	3
The ten largest communities					
Number	10	10	10	10	10
Proportion of all communities (%)	0.3	0.3	0.3	0.3	0.4
Nodes	3,007	5,215	3,730	3,056	2,467
Proportion of all community nodes (%)	16.2	24.5	17.7	17.7	16.8
Edges	5,308	8,903	6,176	4,294	3,990
Proportion of all community edges (%)	18.8	27.6	20.2	17.0	18.4
Holding	2,961	5,138	3,694	3,035	2,447
Gathering	45	73	34	20	19
Market	1	4	2	1	1
